# Getting to implementation: Adaptation of an implementation playbook

**DOI:** 10.3389/fpubh.2022.980958

**Published:** 2023-01-06

**Authors:** Vera Yakovchenko, Shari S. Rogal, David E. Goodrich, Carolyn Lamorte, Brittney Neely, Monica Merante, Sandra Gibson, Dawn Scott, Heather McCurdy, Anna Nobbe, Timothy R. Morgan, Matthew J. Chinman

**Affiliations:** ^1^Center for Health Equity Research and Promotion, VA Pittsburgh Healthcare System, Pittsburgh, PA, United States; ^2^Division of Gastroenterology, Hepatology, and Nutrition, Department of Medicine, University of Pittsburgh, Pittsburgh, PA, United States; ^3^Department of Surgery, University of Pittsburgh, Pittsburgh, PA, United States; ^4^Department of Medicine, Central Texas Veterans Healthcare System, Temple, TX, United States; ^5^VA Ann Arbor Healthcare System, Ann Arbor, MI, United States; ^6^Digestive Disease Section, Cincinnati VA Medical Center, Cincinnati, OH, United States; ^7^Gastroenterology Section, VA Long Beach Healthcare System, Long Beach, CA, United States; ^8^Division of Gastroenterology, Department of Medicine, University of California, Irvine, Irvine, CA, United States; ^9^RAND Corporation, Pittsburgh, PA, United States

**Keywords:** liver, strategies, implementation science, modification, fidelity, hepatology, hepatoma, adaptation

## Abstract

**Introduction:**

Implementation strategies supporting the translation of evidence into practice need to be tailored and adapted for maximum effectiveness, yet the field of adapting implementation strategies remains nascent. We aimed to adapt “Getting To Outcomes”^®^ (GTO), a 10-step implementation playbook designed to help community-based organizations plan and evaluate behavioral health programs, into “Getting To Implementation” (GTI) to support the selection, tailoring, and use of implementation strategies in health care settings.

**Methods:**

Our embedded evaluation team partnered with operations, external facilitators, and site implementers to employ participatory methods to co-design and adapt GTO for Veterans Health Administration (VA) outpatient cirrhosis care improvement. The Framework for Reporting Adaptations and Modifications to Evidenced-based Implementation Strategies (FRAME-IS) guided documentation and analysis of changes made pre- and post-implementation of GTI at 12 VA medical centers. Data from multiple sources (interviews, observation, content analysis, and fidelity tracking) were triangulated and analyzed using rapid techniques over a 3-year period.

**Results:**

Adaptations during pre-implementation were planned, proactive, and focused on context and content to improve acceptability, appropriateness, and feasibility of the GTI playbook. Modifications during and after implementation were unplanned and reactive, concentrating on adoption, fidelity, and sustainability. All changes were collaboratively developed, fidelity consistent at the level of the facilitator and/or implementer.

**Conclusion:**

GTO was initially adapted to GTI to support health care teams' selection and use of implementation strategies for improving guideline-concordant medical care. GTI required ongoing modification, particularly in steps regarding team building, context assessment, strategy selection, and sustainability due to difficulties with step clarity and progression. This work also highlights the challenges in pragmatic approaches to collecting and synthesizing implementation, fidelity, and adaptation data.

**Trial registration:**

This study was registered on ClinicalTrials.gov (Identifier: NCT04178096).

## Introduction

Most clinical practice guidelines and evidence-based practices (EBPs) never reach the populations they are intended to help (1, 2). Implementation science seeks to address this knowledge-to-practice gap through the study of implementation strategies—techniques to enhance the adoption, implementation, and sustainment of evidence-based knowledge to improve population outcomes (3, 4). Implementation strategies work best when they are selected to address contextual implementation barriers and fit with both the EBP and local context (5–8). While taxonomies (9–11) have been developed to classify and standardize the definitions of the dozens of strategies available, it remains challenging for practitioners to effectively choose and tailor these strategies to local clinical contexts (8).

Practitioners desire user-friendly implementation “playbooks”—guidance documents that provide options to tailor strategies for organizational and environmental contextual factors (12, 13). Several process frameworks (e.g., Replicating Effective Programs, Dynamic Adaption Process for Exploration, Preparation, Implementation, Sustainment, and the Tailored Implementation for Chronic Disease) have also been developed to guide researchers and practitioners through the steps of employing implementation strategies to adopt new EBPs (14–19), yet these frameworks can be perceived as complex by frontline practitioners and use academic jargon that make real-world translation difficult without implementation support. Moreover, these frameworks often lack clear guidance on how to efficiently and effectively select and tailor strategies by understanding strategy mechanisms of action (8, 20, 21).

Getting To Outcomes^®^ (GTO) is a 10-step implementation playbook originally developed to facilitate the adoption of EBPs in community settings by building an organization's capacity and empowering users to embrace strong evaluation practices, become results-oriented, and adopt continuous quality improvement methods to select, plan, implement, and evaluate EBPs (22). To guide practitioners through the 10 steps, GTO has three primary multi-component strategies: (1) a manual of resources and worksheets (called “tools”), (2) training for each step, and (3) ongoing technical assistance and facilitation—i.e., the use of outside personnel to support the change in work practices through encouragement, feedback, and action promotion via regular, ongoing meetings (23). Across five quasi-experimental and randomized trials, community settings using GTO gained capacity, implemented their programs with greater quality, achieved better individual participant outcomes, and were more likely to sustain their programs compared to settings not using GTO (24–30). However, GTO has been used regularly in community, not health care settings. In addition, GTO was designed for selection of effective *interventions*, not for the selection of *implementation strategies*.

We aimed to adapt GTO to support implementation strategy selection, tailoring, and evaluation to improve the uptake of evidence-based cirrhosis care in Veterans Health Administration (VA) healthcare facilities. Adaptations and modifications represent changes to form (i.e., the shape and delivery of the strategy) while retaining core function (i.e., purpose of the strategy) (31). Adaptation has been an inexact science, and there is significant need for systematic data collection to capture adaptations for implementation strategies—e.g., *what* modifications to strategies occurred, *who* initiated them, *why* and *when* the modification was initiated, and *how* these modifications affected implementation or clinical outcomes (32, 33). We describe the initial adaptations to GTO's strategies to develop Getting To Implementation (GTI) and subsequent modifications to GTI made as part of a hybrid type III effectiveness-implementation trial (34).

## Materials and methods

### Design and setting

GTI was developed in the context of an ongoing program evaluation, conducted by the embedded implementation science evaluation team for the VA National Gastroenterology and Hepatology Program and the HIV, Hepatitis, and Related Conditions Programs (HHRC). Per regulations outlined in VA Program Guide 1200.21, this project was deemed a non-research operations activity (35). VA employee participation was voluntary. This study was registered on ClinicalTrials.gov (NCT04178096).

The embedded evaluation team worked with facilitators to deliver GTI to 12 VA sites with low uptake of cirrhosis care metrics. Site-level teams typically consisted of nurses, physicians, clinical pharmacists, and quality improvement staff distributed geographically across the US. These 12 sites were cluster randomized to three rounds, with 6 months of facilitated implementation and 6 months of follow-up between October 2020 and October 2022.

**Figure 1:**
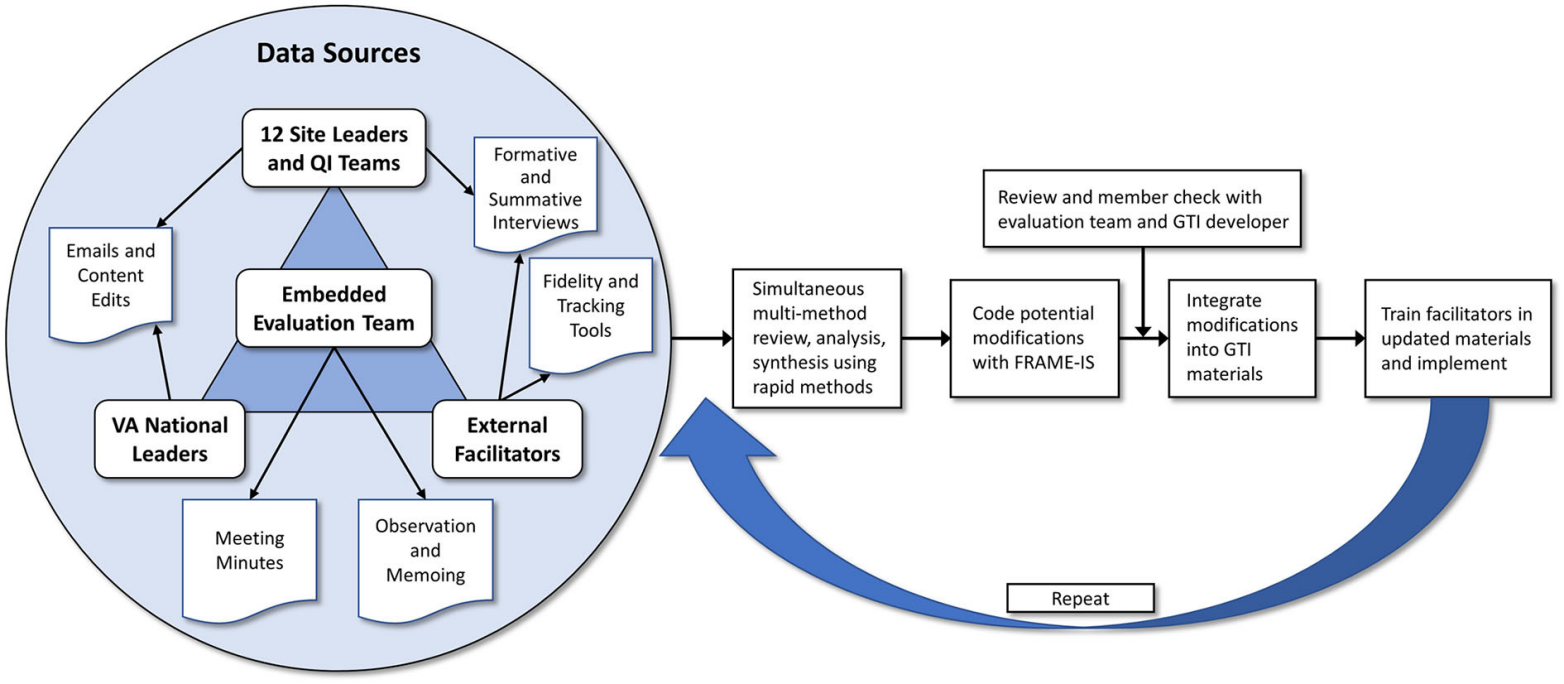
Process to identify, analyze, and integrate modifications to Getting To Implementation.

Changes to GTI were made twice: (1) significant adaptations were made leading up to the hybrid III trial (“pre-implementation”) to transform GTO into GTI and (2) modifications during and after the trial (“post-implementation”) focused on using the experience of the trial to further refine GTI. Our multidisciplinary team of gastroenterologists, implementation scientists, and quality improvement specialists met weekly and agreed upon adaptations and modifications to create GTI for the VA. Figure 1 displays the process to identify, analyze, and integrate modifications.

### GTI intervention

Like GTO, the GTI intervention involves a playbook, training, and facilitation by a two-person facilitator team. The playbook includes a set of steps with tools/worksheets to guide site implementation teams through the GTI process. Training and facilitation involve biweekly virtual calls with two facilitators over a 6-month active implementation period, followed by 6 months with three additional sustainment meetings.

To ensure fidelity to GTI, facilitators were trained in both the facilitation method as well as the GTI process. Initially, the evaluation team, including two team members who would serve as facilitators (“evaluation-facilitators”) were trained to perform facilitation by the Behavioral Health QUERI via two half-day, virtual sessions (36, 37). Evaluation-facilitators were two masters level social workers. Using a train-the-trainer model, the evaluation team then trained three clinician-facilitators via two virtual half-day sessions. Clinician-facilitators included hepatology providers (two advanced practice providers and one RN). Each of the three clinician-facilitators had 50% time devoted to non-clinical quality improvement activities, including GTI facilitation. Another three clinician-facilitators joined the project later and were trained by the evaluation team and the experienced facilitators through two virtual half-day sessions, as well as through shadowing the experienced facilitators. In pre-implementation, facilitators met with the evaluation team weekly to review and practice using the GTI tools. During implementation, these weekly meetings evolved to site-specific progress updates and discussion. All accepted modifications were agreed upon by consensus by the evaluation team and facilitators during team meetings.

### Data collection and analysis

The evaluation team collected data from multiple sources to document GTO-to-GTI adaptations and subsequent GTI modifications, alongside tracking implementation and fidelity process data (38, 39). We measured fidelity to GTI implementation by electronically tracking in Microsoft Excel^®^ the time spent on each step, how and by whom GTI tools were completed, challenges encountered, and other relevant field notes.

All notes from evaluation team-facilitator meetings and facilitator-site team meetings were captured live, and meetings summarized using field notes and ongoing reflections from facilitators and direct observers. Thirteen summative interviews with sites asked about experiences with GTI and facilitation, the core strategies, and any barriers and facilitators experienced. We conducted a review of materials (e.g., emails, instant messages, meeting notes) and tracked GTI playbook and tool changes throughout implementation throughout the course of the trial.

Initial GTO-GTI adaptation and later GTI modification data were coded using the Framework for Reporting Adaptations and Modifications to Evidence-Based Implementation Strategies (FRAME-IS)—including what was modified and the nature of the modification, rationale, timing, level, who participated in the decision, and how widespread it was (Table 1) (32). Two coders (VY, MM) conducted directed content analysis using the predetermined FRAME-IS codes (40). Member checks with a GTO developer (MJC) followed to verify fidelity consistency with relation to the original GTO (41). For each adaptation/modification, we coded its goal using common implementation outcomes (i.e., acceptability, appropriateness, feasibility, adoption, fidelity, reach, and sustainability) (5). For example, adaptations developed during pre-implementation might aim to improve acceptability (i.e., perceptions of fit), whereas adaptations proposed during post-implementation might focus on sustainability (i.e., maintenance or institutionalization of the newly implemented practice). The team, including all facilitators and notetakers, discussed notetaking, coding, and other considerations throughout the course of the study to ensure comparable interpretations.

**Table 1:** Definitions of key implementation outcomes and modifications.

**FRAME-IS coding definitions** **(****32****)**	
What is modified?	**Content, Training, Evaluation**: changes in the subject matter of a strategy or the way implementers are trained or the way a strategy is evaluated
	**Context**: changes to the format, setting, personnel, or population of a strategy
What is the nature of the modification?	Adding, removing, substituting, repeating, etc.
Is it fidelity consistent or inconsistent?	Do the changes reflect a preservation or alteration of core elements
What is the goal?	**Reach**: is the strategy reaching the intended population
	**Adoption**: is the intended population using the strategy
	**Acceptability/appropriateness**: is the strategy perceived as fitting, relevant, or compatible
	**Fidelity**: extent to which implemented as intended
	**Sustainability**: is the strategy integrated into routine practice
What is the level of the rational for modification?	Micro: recipient or implementer
	Meso: organizational
	Macro: sociopolitical
When is the modification initiated?	Pre-implementation, implementation, scale up, maintenance, sustainment
Is the modification planned or proactive?	Planned and proactive: adaptation
	Planned and reactive: adaptation
	Unplanned and reactive: modification
Who participates in the decision? Who makes the ultimate decision?	Recipient, implementer, implementation support, funder, manager, leader
How widespread is the modification?	Individual recipient, group of recipients, individual implementer, group of implementers, organization, network

## Results

GTI facilitators met with the evaluation team 67 times during pre- and post-implementation (2020–2022). External facilitators conducted 169 facilitation meetings with the 12 site teams during the same time period.

### Context adaptations and modifications

Adaptations to GTO were made to address the contextual differences between GTO and GTI settings and improve perceptions around acceptability, appropriateness, and feasibility. Adaptations were coded as relating to (1) setting and population, (2) delivery format, and/or (3) tools.

#### Setting and population

GTO was originally intended to help community members *choose* an EBP; GTI was developed for frontline healthcare workers implementing a *specific* EBP—i.e., cirrhosis care. Thus, pre-implementation adaptations included simplifying the GTO manual and using clinically oriented language (e.g., discussing Veteran patients and clinicians rather than community members and implementers). These adaptations aimed to increase the initial acceptability and appropriateness of GTI through increased perceived fit, relevance, and compatibility. For example, recognizing the hierarchies and team structures at VA sites, we developed recruitment materials to introduce GTI to VA leadership to gain initial buy-in.

Further modifications for fit continued post-implementation based on ongoing discussions with facilitators and the evaluation team. For example, the original GTI manual and slides aimed to teach site teams intricacies of process mapping, a technique to diagram the discrete steps of care to identify bottlenecks and other quality deficits (42). Subsequently, this was simplified to not teach the specific mapping symbols used by system redesign engineers but rather the most essential aspects of mapping out the steps of a clinical workflow. Conversely, some of the examples that were initially included in the slides (e.g., using the analogy of changing ingredients in a cookie recipe to convey the concept of strategy adaptations within GTI) were thought to be *over*simplifications and were omitted to acknowledge complexity and to respect the clinical experience of highly educated clinicians.

#### Format

GTO was originally developed to be delivered and supported by a facilitator. GTI continued to use facilitators; however, facilitator scope of roles and tasks were more clearly tailored to cirrhosis care and timebound. GTI facilitators held biweekly site team meetings for a 6-month period. We developed Microsoft PowerPoint^®^ slide decks to guide facilitator-site meetings through each of the GTI steps. Facilitators and site teams favorably viewed the format of GTI, structured agenda and accessible slides decks. Site participants reported during summative interviews that they would not have benefitted from GTI to the same degree had it not been delivered by a facilitator, yet both site participants and facilitators suggested accelerating the steps and frontloading facilitator support (particularly when sites had an established and engaged team ready to begin GTI).

Once sites completed all GTI steps, a concluding meeting was added to celebrate their “graduation” from GTI. This included extending an invitation for local leadership to join the meeting, a summary document of site progress and completed GTI tools, along with a recognition plaque.

#### Tools

Both GTO and GTI use tools to guide teams. The tools accompanying steps were adapted and further modified and made simpler. Seventeen GTO tools became 13 GTI tools at pre-implementation, which were further reduced to nine tools post-implementation. In terms of tool context, GTI adapted the tools to reflect a more health care system-oriented perspective, rather than that of a community-based non-profit organization. In GTO, sites are asked to attempt tool completion independently and then send to facilitators for review and feedback; tool iteration continues until both the site and the facilitator agree that the tools are of sufficient quality. In contrast, GTI sites completed tools collaboratively with facilitators during meetings. We observed site preferences for pre-populated tools (i.e., known information is already filled in for sites by facilitators prior to the live meeting), which, sites commented, demonstrated personalized attention from facilitators—“it was nice to see that you were listening during prior calls.” Changing to a more collaborative approach to completing tools helped maximize team engagement and discussion.

Sites would refer to and update tools from previous steps throughout the implementation period. Given that team size ranged from a single person to multiple interdisciplinary individuals unfamiliar to one another, the larger site teams required more internal and granular conversation after certain GTI meetings to come to consensus on key decisions, delegating tasks, and action planning. A suggestion from several of these larger site teams that was fulfilled was to allot time at the end of the hour for teams to discuss planning independently from the facilitator.

### Content adaptations and modifications

Content changes are described at the level of each GTI step and were made to improve adoption, reach, fidelity, and sustainability. The most substantive adaptations to GTO content occurred pre-implementation and collaboratively with the evaluation team, facilitators, and GTO developer (MJC). The original ten GTO steps were reordered, integrated, added to, and tailored to produce eight GTI steps during pre-implementation. Based on feedback of teams and facilitators, we further adapted GTI steps after the trial based on summative feedback. Table 2 presents changes to GTO and GTI steps and tools over time.

**Table 2:** GTO to GTI adaptations and modifications over time.

**GTO Original**		**GTI Pre-implementation**		**GTI Post-implementation**	
**Steps**	**Tools**	**Steps**	**Tools**	**Steps**	**Tools**
0. Planning and preparation		0. Planning and preparation, build a team	**0.1 Team development**	**1. Build a team and identify current processes**	**1.1 Process mapping**
			0.2 Completion calendar		**1.2 Team development**

1. Problem identification	1.1 Data catalog	1. Identify gaps and goals	1.1 Evidence-based practice	2. Establish goals	2.1 Evidence-based practice
	1.2 Community resources assessment		**1.2 Process mapping**		
	1.3 Triaging among problems				

2. Identify goals and desired outcomes	2.1 SMART desired outcomes	**2. Assess facilitators and barriers to implementation**	**2.1 Workflow barriers**	**3. Assess and prioritize strengths and barriers**	**3.1 Strengths and barriers assessment**
	2.2 Community action plan		**2.2 Facilitators and barriers assessment**		**3.2 Barriers prioritization**
			**2.3 Importance difficulty matrix**		

3. Find existing programs or best practices worth adopting	3.1 Evidence synthesis	**3. Choose implementation strategies**	**3.1 Choosing your strategies**	**4. Choose solutions**	**4.1 Choosing your strategies**

4. Modify the program or practices to fit your needs	4.1 Fit assessment	**4. Adapt strategies and address readiness**	**4.1 Readiness to use an implementation strategy**	**5. Plan and adapt solutions**	5.1 Work plan
	4.2 Culturally appropriate checklist				

5. Assess capacity to implement the program	5.1 Readiness to implement				

6. Make a plan for getting started	6.1 Work plan	5. Plan implementation	5.1 Work plan		
	6.2 Budget				
	6.3 Process evaluation planner				
	6.4 Outcome evaluation planner				

7. Track planning and implementation	7.1 Process evaluation results summary	6. Implement and evaluate	6.1 Implementation tracking	6. Implement, evaluate, and improe	6.1 Evaluation and improvement

8. Evaluate the program's success	8.1 Outcome evaluation results summary				

9. Continuous quality improvement	9.1 Continuous quality improvement	7. Improve implementation	7.1 Continuous quality improvement		

10. Sustainment	10.1 Sustainability review	8. Sustain implementation	8.1 Sustainability review	7. Sustain and look ahead	7.1 Sustainability and review

Bold denotes new to GTI.

#### Team building

The original GTO manual suggests developing a team that mixes frontline staff who are directly responsible for conducting the EBP and managers who have the higher-level authority to make decisions involving resources (primarily staff time). However, GTO had never codified this suggestion into a formal GTO step. Thus, recognizing the importance of teams in implementation efforts, GTI created an official team building “Step 0.” The accompanying tool delineated the process of setting up and managing a multidisciplinary team, and seeking leadership buy-in. To further encourage site and team accountability and engagement, we developed a site agreement letter, which outlines expectations of what the facilitators would provide as well as the expected role of the partnering site. In practice, some sites and facilitators perceived Step 0 as too long, while other sites extended the step of developing a team to ensure sufficient recruitment of site team members. Although we had already formally included the process of developing the team as a step, we further clarified its importance by renaming it from Step 0 to Step 1 at post-implementation.

#### Goal setting

GTI consolidated several early GTO steps to simultaneously identify problems, gaps, and goals. This adaptation reflected how clinical quality measures and guidelines are usually pre-determined and/or set by leadership. In GTO, sites start in a general content area (e.g., underage drinking, teen pregnancy) and conduct a needs and resources assessment to learn more about the drivers of the overall problem, and then come to a consensus on which aspects of the problem to tackle (e.g., abundance of bars, contraception not readily available). Ultimately, this step's function was augmented from educating, raising awareness, and leading through a decision process to a focused goal-setting function while retaining the basic form of the steps.

#### Context and barrier identification

The most significant change between GTO and GTI was the creation of two entirely novel steps and their accompanying tools to improve the adaptability, fit, and feasibility of GTI in the VA setting. The function of GTI's Step 2 is to identify implementation barriers and triage them to choose the priority barriers to address. The GTI Strengths and Barriers Assessment Tool includes implementation determinants from the Consolidated Framework for Implementation Research (43). The tool includes 23 of 39 constructs, omitting constructs less relevant to the VA and cirrhosis care setting. The tool is first completed by individual team members; each member responds to a prompt (i.e., “Clinicians believe the evidence behind surveillance is strong”) on a Likert scale ranging from 1 = “Strongly disagree” to 5 = “Strongly agree.” The tool is then discussed among the entire team with facilitators during a site meeting to arrive at a consensus on a score—i.e., team members eventually all agreed on a score even if they initially may not have. This step continued to be refined during implementation because it was consistently problematic in terms of flow and understandability during round one. We revised the language in the Strengths and Barriers Assessment Tool and adjusted the scale, removing a neutral response option to avoid frequent decision ambivalence among site participants.

In GTI, a process mapping activity was added to assess new forms of barriers by creating a visual depiction of the points of the clinical workflow to help uncover bottlenecks and other barriers. However, at post-implementation we determined process mapping would be more beneficial at the earlier team development step to identify possible team members throughout the workflow improvement process. The process map also remained as a reference point in the barrier step.

Once workflow and organizational barriers were identified by sites, barrier prioritization involved additional tools and discussion. In round one, the Triaging Barriers Tool was experienced as too broad and rudimentary to be helpful in translating all identified barriers to priority barriers. Therefore, we substituted the tool with an Importance-Difficulty Matrix Tool to categorize barriers more concretely and identify issues of prospective fit. Then during post-implementation, we further refined the prioritization process and transitioned to using the importance-difficulty matrix to guide completion of a new Barriers Prioritization Tool which incorporated the concept of leveraging identified strengths to address barriers rather than solely focusing on challenges.

#### Selecting strategies

GTI Step 3 is another novel step and involves an empiric approach to choosing implementation strategies in the context of barriers identified in the previous step. This step is entirely distinct from GTO, which focuses on choosing an evidence-based intervention and not implementation strategies.

The eight effective strategies embedded in the GTI playbook were developed through a multi-step process previously described in detail (44–46). Briefly, we fielded surveys in two consecutive years to identify implementation strategies being employed across all VA sites. We then identified strategies associated with positive cirrhosis care outcomes using correlational and configurational methods. The evaluation team then interviewed survey respondents and other providers at higher-performing sites to operationalize the subset of effective strategies. Finally, we integrated the effective strategies into the GTI playbook (47).

In the pre-implementation phase, the GTI manual specified eight strategies that were found to be empirically associated with better outcomes in our previous survey work were labeled as “High Value” strategies (47). We also created a tier system to set apart three of the “High Value” strategies from the other five based on the strength of the empiric relationship to cirrhosis care. During implementation we refined strategy descriptions and changed the labels from “High Value” to “Core” to remove the distinction between the two tiers of strategies.

Each of the core implementation strategies includes an accompanying appendix to aid in operationalizing it, and a tracking form to document use and fidelity. Although the appendices with core strategy details were intended for thorough site review, facilitators reported a perception of minimal engagement with these more comprehensive materials. Instead, sites relied on live facilitator discussion and slide materials. Another modification was that one of the core strategies, Plan-Do-Study-Act, was subsumed/integrated into the GTI steps 4–7 as a central part of continuous quality improvement rather than retained as a standalone strategy.

#### Planning and adapting strategies

GTI combines and simplifies two GTO steps and focuses on adapting/tailoring the core strategies from GTI Step 3 to the context defined in Step 2. GTO's Step 6 involved planning the intervention, budgeting, and preparing for process and outcome evaluation. We disassembled the step to focus on planning the implementation strategy rather than the intervention. The new GTI step concentrating on the concept of fit and adapting core strategies was conceptually challenging for some site participants as well as facilitators. Furthermore, considering adaptation prior to planning the work was perceived as incongruent with real-world implementation. In response, and to improve acceptability and appropriateness of GTI, we reversed planning and adapting steps, clarified narrative text, and refined the tools. In post-implementation, we further simplified and consolidated these two steps into one to simultaneously plan and adapt strategies. We removed the Readiness to Use an Implementation Strategy tool because it was originally intended for the eight core strategies individually, but sites assessed readiness more holistically in earlier steps or did not use the tool altogether.

#### Implementing and evaluating strategies

The next three GTO steps centered on implementing and evaluating were first collapsed into two during pre-implementation. Given GTI's difference from GTO on the predetermined EBP, it was possible to prepopulate evaluation questions, such that clinical and operational implementers did not have to *de novo* develop the evaluation as in GTO. GTI used an audit with feedback strategy to monitor process and outcomes, unlike in GTO where individual sites are generally responsible for their own data collection, with support from facilitators. This adaptation was consistently well received by sites; however, the success of this step might not have been possible had an existing population health management tool not been in place. The VA's pre-existing cirrhosis dashboard with automated reports was the main source of performance data and accessible to implementers (48). Post-implementation, we further consolidated the implementation and continuous quality improvement steps into one cohesive implement-evaluate-improve step because both facilitators and sites felt the content was duplicative across steps.

#### Sustaining strategies

GTO was developed for program implementation (with a defined start and end), while GTI was adapted for a continuous clinical process. Accordingly, a “sustainability check” that encouraged sites to consider sustainability early and often was included in every GTI step. Even so, participants reported that getting strategies implemented was not sufficient to maintaining them as priorities at the site level. Thus, GTI's step on sustaining implementation was believed to be necessary but potentially unreachable. Per some sites and facilitators, the sustainability step came too early after implementation began and was thus postponed until the 3- and 6-months post-implementation meetings. In addition to the step's improper timing, one facilitator reflected, “a lot of the discussion was not new, though a few new or evolving ideas and adaptations came from use of it.” Nevertheless, given GTI's focus on applying and developing continuous quality improvement methods, the final step was retained with minimal adjustments post-implementation.

### Training adaptations and modifications

GTO training is typically delivered to community organizations by a single facilitator and in-person facilitator training is up to 16 hours in duration. To improve feasibility during COVID travel restrictions, GTI facilitator training was shortened as two 3-hour blocks of virtual training with and edited GTO slide deck role-playing and modeling training exercises created to accompany the didactic training. Three clinician-facilitators who joined the trial during round 2 benefitted from shadowing facilitators in practice before leading facilitation in round 3. Facilitator feedback on the thoroughness of the training was universally positive. However, several facilitators sought more detailed descriptions of theory and application to ensure they could help site recipients with interpretation of the GTI process. For example, we added a table to depict barriers from one step may be linked to core strategies in another step.

### Measurement and evaluation modifications

Two evaluation-facilitators were responsible for all facilitation and fidelity tracking with meeting note support from two research assistants (SG, MM). Facilitators reflected on the considerable burden of tracking implementation and fidelity, and often, desiring more efficient and less intensive procedures to capture this process data. Facilitators found it most onerous to estimate time devoted to preparing emails and other unstructured support and cautioned that effort was likely an underestimate.

Notably, facilitators proposed and enacted effective methods for deduplicating data sources, and saving and organizing content (e.g., email correspondence, completed tools). One evaluation modification after round one included simplifying the facilitation tracking sheet by deduplicating fields already being collected in the GTI fidelity tracking form, and adding new summary fields to note the barriers, facilitators, strategies, or adaptations discussed and follow-up tasks. Still, facilitators sought more pragmatic methods to collect implementation, fidelity, and adaptation data, resulting in further cosmetic and organizational modifications to reduce the burden of tracking.

## Discussion

We developed an adapted implementation playbook called “Getting To Implementation” and described further modifications made to meet the needs of practitioners in real-world health care settings. The study of adaptation is ideally suited for participatory research settings such as ours in VA where researchers and operational partners work in close collaboration and are heavily invested in the co-design and evaluation of implementation efforts.

Often, implementers receive little guidance about selecting strategies to support operationalizing complex clinical practice guidelines implementation (49). Our GTI playbook is a curated seven-step improvement process to support strategy selection, tailoring, and evaluation in cirrhosis care (50). Adaptations to GTO were made to improve the fit with clinical rather than community-based workflow, language, and culture. Our multi-method and multi-perspective approach allowed for often unarticulated needs from diverse perspectives to become part of the design process. Based on feedback from our partners, we formalized the team building step, simplified context assessment, specified potential links between context and strategy selection and adaptation, and integrated implementation and evaluation.

FRAME-IS is a highly practical in-depth coding system that was critical for tracking adaptations to GTO and modifications to GTI. Still, the science of conducting and measuring adaptations in implementation science is nascent and this growing area of empirical inquiry demands more attention. Ongoing discussions throughout trial implementation helped develop a set of heuristics to designate what constituted a significant modification to form and/or function to enhance contextual fit or clinical outcomes vs. non-significant modifications. A question remains about the transparency of adaptation, modification, tailoring and the level of granularity required in tracking changes. Adding explicit reflection on form vs. function in the FRAME-IS could enable deeper understanding of mechanisms throughout adaptation and modification of strategies. Greater attention to the goals of modifications and their earlier consideration might permit more thoughtful and purposeful deliberations on changes. Nonetheless, the decisions that were captured yielded valuable information to improve GTI usability.

Our pre-implementation adaptations included planned proactive and fidelity-consistent changes to GTO across all areas—training, materials, delivery, context, content, materials, and evaluation. A closer look shows these adaptations focused on shifting functions from motivation of implementers in GTO to capability of implementers in GTI (51). Our empirically informed modifications included the voices of different partners throughout the health system and were critical to form adjustments while maintaining core functions. This “relationship-centered” (52) rather than individual-centered design thinking approach was essential to our study.

Despite some flexibility with tracking minor changes, real-time tracking of adaptations and modifications is burdensome and time intensive. Alternative approaches using pragmatic, efficient, and periodic methods are needed, while taking care to track granular changes over time. Continued innovative thinking and translation of other industrial engineering methods may both lessen the burden of tracking and improve the science (53). Importantly, as done in this study, ongoing and mid-course rather than solely *post-hoc* evaluation of modifications is needed to capture complete information and glean insights (54).

While developed to improve VA cirrhosis care, GTI's “choose your own adventure” approach is amenable to clinical area specification or customization, while maintaining generalizability. Modification for scaling and spreading GTI continues within VA, in tandem with the larger field of evidence-based quality improvement expanding (55, 56). As the largest integrated healthcare system in the US, VA trains much of the healthcare workforce. Deploying simple implementation playbooks serves the purpose to educate the next generation of healthcare professionals and leaders in methods that are rigorously developed, acceptable and applicable.

### Strengths and limitations

This study is not without limitations. Although we had a robust multi-method tracking and evaluation approach, adaptations occasionally remained difficult to comprehensively capture. For example, as facilitators have some degree of delivery autonomy and are expected to tailor to the current situation, those more subtle modifications may not have been captured (57). Also, due to multiple trackers and note takers, measurement consistency could have impacted the findings. To mitigate this limitation, trackers met continually to discuss the processes of tracking data and their interpretation. In cases where there was a disagreement, a member of the evaluation team (VY) adjudicated differences. A study strength was tracking adaptations in real-time and longitudinally throughout the course of the study to understand local modifications.

Future work will examine fidelity to the GTI model, predictors of fidelity, and associations with cirrhosis care and outcomes. In addition, while the opportunity to suggest effective implementation strategies based on actual data was a strength in this current project, other efforts might not have that kind of strategy data available to embed in GTI. Thus, future work, using large, previously collected data sets and machine learning algorithms would be useful in these situations to optimize strategy selection for a particular improvement project.

## Conclusion

Implementation playbooks can support intervention adoption and sustainment. This article detailed the process of the initial adaptation at pre-implementation, followed by modifications post-implementation. Adapting GTO into GTI required simplifying GTO and making it more practical for a clinical audience. As embedded evaluators using a pragmatic approach, we were able to share and act upon feedback quickly, learn, and iterate GTI through a participatory co-design process. This work contributes to the growing base of methods to help frontline staff and organizations plan for and promote the uptake of EBPs.

## Data availability statement

The raw data supporting the conclusions of this article will be made available by the authors upon reasonable request.

## Ethics statement

Ethical review and approval was not required for the study on human participants in accordance with the local legislation and institutional requirements. Written informed consent for participation was not required for this study in accordance with the national legislation and the institutional requirements.

## Author contributions

SSR and MJC initially conceptualized the study. VY, CL, BN, DS, HM, AN, MM, and SG contributed to data collection. VY performed data analysis. VY, DEG, MJC, and SSR wrote the original manuscript draft. All authors contributed to the conception and design of the study, performed critical review of the manuscript, and approved the final version.
